# Is bad news on TV tickers good news? The effects of voiceover and visual elements in video on viewers’ assessment

**DOI:** 10.1371/journal.pone.0231313

**Published:** 2020-04-15

**Authors:** Konrad Maj, Stephan Lewandowsky

**Affiliations:** 1 Department of Psychology, SWPS University of Social Science and Humanities, Warsaw, Poland; 2 School of Experimental Psychology and Cabot Institute, University of Bristol, Bristol, England, United Kingdom; Ghent University, BELGIUM

## Abstract

In our experiment, we tested how exposure to a mock televised news segment, with a systematically manipulated emotional valence of voiceover, images and TV tickers (in the updating format) impacts viewers’ perception. Subjects (N = 603) watched specially prepared professional video material which portrayed the story of a candidate for local mayor. Following exposure to the video, subjects assessed the politician in terms of competence, sociability, and morality.

Results showed that positive images improved the assessment of the politician, whereas negative images lowered it. In addition, unexpectedly, positive tickers led to a negative assessment, and negative ones led to more beneficial assessments. However, in a situation of inconsistency between the voiceover and information provided on visual add-ons, additional elements are apparently ignored, especially when they are negative and the narrative is positive. We then discuss the implications of these findings.

## Introduction

The simultaneous display of multiple messages that provide a substantial amount of information has become a common practice in electronic media. It can be observed mainly in the news, but also in weather, business, or sports programs. Contemporary TV news programs present significant amount of data, which is updated on screen very quickly [1]. Audiovisual communication is typical of contemporary electronic media: information is presented in both visual and auditory form [2–5]. At the same time, due to the competition on the media market, we can observe a large number of methods aimed at attracting viewers’ attention and making the media more attractive [6]. In consequence, simultaneous broadcasting of various information by means of parallel channels (e.g., audio and video) has become increasingly common, even though it can significantly lower the fidelity of processing [7].

Media news play a special role in political messaging [8]. Research by McCombs and Shaw [9] showed that a correlation between what the media are interested in and what is important for voters is as high as 0.97. From using images to influence viewers’ cognitive and emotional reactions [10] to developing attitudes and concepts about candidates for a particular political position [4, 11–16], the media impacts our final election decisions [17].

### Audiovisual elements in the modern media: Images and TV tickers

In the world of the media, images attract attention effectively both in the case of traditional media [18, 19] and the Internet [20]. Images enhance a message and make it more salient [5, 14, 21]. For that reason, news producers enrich media communication with graphical elements in order to encourage viewers to follow information and to improve its reception [1, 22–26].

Additional visual elements can influence the evaluation of material by a variety of means. For example, from a classical-conditioning perspective, an attitude towards an object can be transferred from the associating stimulus [27]. This phenomenon is often used in advertising [28–30]. That is why many brands are promoted by attractive, liked and happy persons (especially media icons), and why so many products are presented in a desirable, pleasant context (e.g., nature, the beach, sun, laughing, playing, etc.).

Research shows that if a story is accompanied with still or moving images, this increases the memorization of its content [31, 32]. Images support the main media with an emotional element and that is why they can significantly influence the interpretation of the message conveyed through the verbal channel [33]. Mitchell and Olson [34] showed that photographs are not received by means of a purely cognitive path. Verbal elements enriched with visual items are persuasive in two ways: the verbal message evokes beliefs, while nonverbal aspects (e.g., photos) influence emotions [35]. It has also been shown that if an article on a particular topic is accompanied by a tendentious photograph, it strongly influences attitudes and this effect can last for several days [36].

Broadcast media have recently begun to rely on TV tickers. This graphical element is presented (usually) at the bottom of the screen in order to demonstrate additional information—data which may potentially be of interest to a viewer (e.g., the weather or stock market information) or which supplement the current coverage. Tickers are also a good way of presenting breaking news without interrupting the ongoing video material. That is perhaps why the age of regular information tickers started in 2001, during the World Trade Center attacks [37]. At the time, CNN wanted to communicate all pieces of news and present video material simultaneously. In Poland, a turning point in this respect was in April 2005, when two channels, TVN and TVP, were giving an account of the final days of Pope John Paul II. Nowadays, information tickers are commonly used by news stations all over the world.

Each station has its own pattern of providing information on the ticker. Tickers are present on the screen permanently or temporarily. Two main types of ticker can be identified—one to present common information, and another to present breaking news.

Information tickers can encourage people to follow news, and they can resemble headlines in newspapers—they may also constitute complete or self-sufficient information [38]. Research on students shows that almost 85% of them use tickers as their source of information, while 78% read tickers because they have no time to follow the whole material or consider such abbreviated information sufficiently enough to summarize particular events [39].

There are, however, few studies that have specifically examined the effects of TV tickers. So far, researchers’ attention has been focused on the effectiveness of information tickers in understanding and memorizing information in the context of multi-tasking [3], [40] reception of specific designs of tickers [2], display style (updating or scrolled format) [37], or as a promotional tool [41, 42]. In one of the first series of experiments, news presented on the screen in graphic form or in the form of news tickers was found to reduce the understanding of information in both the auditory and visual channel compared to no visual condition. Information tickers may therefore divide viewers’ attention, impairing information processing by negatively influencing the content of presented information in the whole message [3].

Therefore, we believe that additional visual elements added to the media coverage influence the attitudes of viewers, shaping them toward the content of these additives. Hence the hypothesis:

H1. Additional visual elements (images and tickers) in a video material will influence viewers’ attitudes toward a politician according to their content, in such a way that visual elements with negative content will lower the assessment of the politician, whereas those with positive content will raise it.

### The role of images versus text in political assessments

Visual messages can dramatically change attitudes towards a particular political candidate. During the famous debate between Kennedy and Nixon, it turned out that the recipients who listened to it on the radio were sure it was Nixon who won, whereas viewers watching the debate on TV believed that Kennedy was the unquestionable winner. When analyzing this phenomenon, Druckman [43] showed that viewers who saw this debate on TV were guided by the candidates’ personal traits in their evaluation more than those who listened to the debate on the radio.

Similarly, Coleman and Banning [13] analyzed the US presidential campaign in 2000. It turned out that the television stations presented much more positive nonverbal behavior of Al Gore compared to the behavior of George W. Bush, and those who watched this biased coverage had electoral preferences consistent with this media message.

People shape their first impressions on the basis of image, which translates into the number of votes obtained by a politician in the election [44]. Voters infer competences of a person generally from the face, and this process allows the prediction of election results [45, 46]; unfavorable photographs can significantly influence the judgment of politicians [4, 16] and the final decision to support them in the elections (e.g. [11, 17, 45, 47–49]).

Research on the reception of visual elements on TV carried out by [50] using an Eye Tracker showed that viewers focused much more on the central part of the screen than on the tickers displayed at the bottom (fixation time 41.0% vs. 15.3%), hence it seems that the effect of additional images should be stronger than the tickers.

Moreover, although the impact of TV tickers, compared to images, on attitudes towards politicians has not been tested so far, given that the information tickers contain text, we think it may have a weaker impact on viewers’ judgments, hence our hypothesis:

H2. Images will have a stronger effect on the assessment of a politician than TV tickers.

### Valence of information in political assessment

Positive information about a politician increase their popularity [51], thus also raising the likelihood of people voting for the candidate [52]. However, people tend to focus more on negative information [53–55]. In formulating the assessment, they treat negative information as more important [56] and credible [57, 58] as this kind of data is better remembered [59] and has a stronger impact on attitudes than positive information [60, 61]. The negativity effect is revealed strongly in the reception of media coverage [62, 63]. It has been shown that negative images focus more attention and evoke higher emotional arousal than positive ones [64]. For instance, pairing negative messages with fear-evoking images is an effective way to influence political opinion and voters’ preference [65]. Negative coverage also produces in viewers a much stronger psycho-physiological response than positive information [63]. Therefore, considering the results of previous studies and analyses, we hypothesize that:

H3. Additional visual elements with negative content will have a stronger (negative) impact on the assessment of a politician than positive ones.

### Reception of inconsistent audiovisual information

Analyses by Maria E. Grabe and Erik P. Bucy [14, 66] of audiovisual material presented on television news have shown that very often visual information is inconsistent with the verbal message accompanying it. Incongruent content in visual and other materials can attracts viewers’ attention [67], and enliven the message [68], but ultimately significantly weaken its effect and burden attention [69]. Meta-analysis has shown that the presence of audio and video tracks in media communications in half of cases hinders and in half of cases fosters remembering information [70]. Therefore, whether a visual message will be better remembered when accompanied by a video track or without one, depends on a number of factors. The similarity between content in both channels is of great importance here. If the range of similarity is high, less data can be lost, as some gaps can supplement each other, which could give rise to a supportive role in information processing [71]. But cluttering the screen with tickers and other graphic elements, if they do not complement the main message, may cause an interference effect [2].

In the case of audio-video messages, a video track has a certain advantage over an audio track since it manages attention to a greater extent than audio, but it also significantly influences it (e.g. [69, 72, 73]). Visual stimuli in the media dominate over textual and audio stimuli [74, 75], and can also distract from audio channels [22, 76].

Research on media communication has shown that people rely more on what is written than on what is said by the reporter [23]. In an experiment, college students remembered more details of the same story presented in a newspaper article than of that in the form of an audio or video recording in which the content was read by a reporter [77]. Also, when making a judgment, people rely more on what they see than on what they read or hear [14]. Pictures and images are more likely to be remembered than words, which is well documented in scientific literature and called the “picture superiority effect” [78–83]. In an experiment [84], the degree of memorizing a particular story transmitted in an audiovisual, audio and written form (a transcription of the audio track) has been compared. It turned out that the last case was found to have the greatest efficiency. Thus, there is evidence to suggest that the audio form can lose in a competition for attention with elements displayed on screen.

It has been shown that when people read newspaper articles, they rely on data presented in a photograph, even if the implications of the picture are not confirmed by the neighboring text [85, 86]. Following the results and hypotheses formulated by Boomgaarden et al. [87], we hypothesize that visual elements will have a greater influence on adopting an attitude by subjects than an audio track would (voiceover). Especially if there is inconsistency of data between narration and visual additives, people will be guided by information presented in the form of images and tickers. Thus, the following hypotheses:

H4. In the case of a content inconsistency between voiceover and visual add-ons, images will have the strongest effect on formulated judgments, while tickers will be less influential, and voiceover will be the least powerful in this regard.
H5. In the case of a content inconsistency between voiceover and visual stimuli, subjects will prefer images over tickers and voiceovers as a source of information.

### Research problem and the meaning of the study

In traditional studies on the influence of images of politicians, researchers have generally focused on the impact and role of photographs in the press (e.g. [11, 87]).

Moreover, it appears that in the research conducted to date, only existing television material has been used [37, 40–42, 88]. It should be noted, that not being able to fully modify the original video material hampers the ability to control a number of significant aspects of the coverage and restricts the possibility to create conditions for comparing the response to the TV material with different visual elements (tickers versus images versus control group).

Regarding the content of the information conveyed on the tickers with different affective valence, this kind of influence on the viewers’ perceptions of the person presented in the video has not been verified so far. However, it should be noted that in these studies we do not focus on emotional "breaking news" tickers, but rather on "updating" format tickers [37], i.e. those that appear for a short time in the video material and disappear.

Yet, despite the powerful impact of visual elements, research on TV tickers is lacking and begs further investigation, compared to images and their effects—in particular with regard to the assessments of politicians. It seems that the effects of displaying visual elements of different valence (positive / negative) while simultaneously transmitting the audio message are still unknown. There are also no studies showing whether viewers prefer, and better remember, data from additional visuals or voiceovers. It is interesting and worth investigating whether the viewer’s opinion will be guided more by what a channel’s audio or video is communicating. It is possible to do so when these channels broadcast different (contradictory) content. Very few studies have tested a comparison of conditions with parallel transmissions of conflicting messages using different modalities, e.g. [89].

Thus, the present study is aiming to fill the existing gap.

## Method

In our 3x4 experiment, we tested the reception of a fictitious politician presented in the video material. The basic material had a negative, positive, or ambivalent voiceover commentary toward the politician and in some conditions, we added TV tickers or images of positive or negative content.

Our laboratory protocol is available here:

http://dx.doi.org/10.17504/protocols.io.bau3ieyn .[PROTOCOL DOI]

### Ethics statement

The research project was approved by the Ethics Committee of the Psychology Department at the SWPS University of Social Sciences and Humanities in Warsaw (Poland).

All subjects provided written informed consent and were fully debriefed at the conclusion of the experiment. The opinion number is registered at the university as 8/2015.

### Subjects

The subjects were students of one of the Polish universities (N = 603; 390 women, 213 men, M = 25.44 years, *SD* = 7.15). (None of the demographic variables had an effect on the results, and we therefore do not consider them further.) The respondents were recruited through posters displayed at the university and through personal requests by recruiters on the campus. All subjects were Polish-speaking. None of them declared serious problems with hearing or vision. For each experiment, participants received credit points and vouchers for a free drink (coffee or tea).

### Stimulus material

#### Video material

The experiment employed specially prepared video material, recorded and edited by professionals working for one of the local Polish TV stations. The material lasted 2.5 minutes. In order to retain all the attributes of real material, we placed the logo of a fictional TV station (“Regionalna TV”) in the upper right corner, and at the beginning of the coverage, we also inserted professional graphic credits (“Political roulette”) with inscriptions about the authors of the footage at the end. A professional journalist narrated the video off-camera. The main protagonist, a male politician, was presented in various scenes in the video (he was sitting at his desk and talking on the phone, walking down the street, etc.), but he did not speak at all. There were also three other people who spoke about the politician in the video using fictitious names (two men and one women).

A small town about 250 km away from the study location was chosen as a film set The material used a fictitious city name without indicating its location or other details. The narrative layer of the film concerned the fictional politician, who was running for mayor in a local election. The material was recorded to resemble a short TV reportage. The film touched on the issues of competence, morality, and sociability of the candidate.

#### Experimental manipulation in the video material

*Emotional valence of the voiceover*. The voiceover had negative, positive, or ambivalent content toward the politician (that is, they described the politician as being neither high nor low on the particular features). *Visual add-ons*. Visual elements that included positive or negative content in the form of either tickers or images (a concurrent combination of tickers and images was not employed) were added to the video.

The design of tickers began with creating a set of expressions that were supposed to sound strong and unambiguous (e.g. bad vs. good student, disloyal vs. loyal politician), and then their counterparts in the form of photos were matched.

The content of the tickers concerned the competence, morality, and sociability of the main protagonist. Hence, depending on the condition, the following information was displayed on the tickers: *a weak sportsman* vs. *a good sportsman*, *a bad student* vs. *a good student*, *a disloyal politicia*n vs. *a loyal politician*, *a neglectful frien*d vs. *caring for a friend*, *an irresponsible fathe*r vs. *a responsible father*, *unhelpful neighbor* vs. *helpful neighbor*. The tickers were in an “updating ticker” format, and were therefore displayed for a short time (about 5 seconds) and then disappeared from the screen. The duration of displaying all our visual elements was similar for each image and ticker.

In the image conditions, the tickers were replaced by images. Images were selected as follows: Initially, 8 sets of thematic tickers with negative and positive content were created, which referred to dimensions of competence, morality and sociability.

After conducting a preliminary selection of photos from public databases, photo proposals were selected for each ticker. Pictures were grouped into sets for each ticker in a positive and negative variant. There were 8 sets of tickers. For each individual set, 5 pairs of photos were added (technically resemblant photos were paired, with similar colors and context as well as number of objects in the photo) expressing positive and negative content (e.g. a photo of failed sportsman lying on the ground and a photo of a smiling sportsman raising his arms in a triumphant gesture in an alike context). Then, two independent coders were given the task of assessing and matching to each set of the tickers the most fitting photo sets in terms of content and evoked emotions. Coders were told exactly what to do and were given an example of a similar task to check if they understood instructions correctly. The coders on the sheet were to assess the sets of photos in terms of whether they fit the sets of tickers (on a scale of 1—very poor match, 5—very good match). Then, on this basis, they were to indicate the 2 most suitable combinations for each ticker. In the case of 2 tickers, the indications of pictures turned out to be divergent, so they were eliminated. Hence, the focus was put only on six tickers. Matching photos were chosen and were selected by both coders. In general, Intra-rater reliability was 80% (Cohen's kappa coefficient—0.59).

Visual add-ons were inserted into the film track in such a way as not to disturb the reception of its film content, i.e. in longer, less important scenes of video material.

Thus, each video with different emotional voiceovers (positive, negative, ambivalent) was enriched by additional graphic elements: positive content tickers, positive content images, negative content tickers, and negative content images.

In the control groups, the subjects watched the video without any additional visual elements (but with various voiceovers). In this way, 15 types of films were created (see Table 1).

**Table 1:** Variants of video material used in the study.

Voiceover	Tickers	Tickers	Images	Images	No visual additives
Negative	Positive	Negative	Positive	Negative	
Ambivalent	Positive	Negative	Positive	Negative	
Positive	Positive	Negative	Positive	Negative	

### Procedure

The subjects were randomly assigned to 15 research conditions, of which 3 served as control material against which the results of the others were centered. They were asked to wear a headset and watch a movie about a local politician, and the video was displayed on the computer screen. Subsequently, they filled out a questionnaire containing a number of measures.

### Measures

*Assessment of the politician*. The main component of the questionnaire used in the study was a tool to measure the three dimensions of social perception: competence, morality, and sociability (Cronbach’s alpha obtained: .911, .931, .935). This 3-component structure has been used in the latest studies on social perception [90–94] including perception of politicians, for example: candidates for presidency of the French Republic in 2012 [95] or politicians in Latin America [96]. The scale contained a set of adjectives related to the dimension of competence (e.g., effective, competent, intelligent), morality (e.g., honest, trustworthy, fair), and sociability (e.g., friendly, kind, supportive, sociable). Each feature was rated on a scale from 1 to 7 (where 1 means “definitely not” and 7 “definitely”). Large pairwise correlations between these three dimensions (r (602) = .79 - .87, *p* < .001, *α* = .93) led to the presentation of results as one dimension in the analysis where higher scores mean a better perceptions of politics.

*Reception of a person on dimensions related to visual additives content*. We asked subjects to express an assessment of six semantic differential scales from 1 to 7. “Rate how you generally received Mr. Grzegorz Madej on the following dimensions. Put an X in the appropriate field.” We used dimensions related to the content with visual additives such as: *a weak sportsman* vs. *a good sportsman*, *a bad student* vs. *a good student*, *etc*.

*Centering results and research plan reduction*. Because we were interested in changing the policy assessment with respect to individual research conditions in order to create a dependent variable for the analysis, the attribute ratings were centered on an average level. See the manipulation check section. The research plan was thus limited to conditions of 5 x 3 to 4 x 3 subgroups.

## Results

### Manipulation check

As predicted, in the neutral condition (no visual additives; last column in Table 1), negative voiceover triggered the lowest rating of the politician (M = 2.72, *SD* = .77), ambivalent voiceover gave rise to a moderate rating (M = 3.97, *SD* = .85), and a positivevoiceover improved the reception of the politician (M = 4.69, *SD* = .97), *F* (2, 118) = 52.97, *p* < .001, *η*^2^ = .473, differences between all groups (here and below) were significant at the *p* .001 level.

In order to check the impact of emotional valence of voiceover (negative vs. ambivalent vs. positive) and visual elements added to the video material—the type of stimuli (negative tickers vs. positive tickers vs. negative images vs. positive images) on rated attributes of the politician, a mixed 3 x 4 (valence x stimuli), ANOVA was conducted. The variances in all comparable groups were homogeneous. To create a dependent variable for the analysis—attitude change indicator, the attribute ratings were centered on an average level in three conditions in which subjects saw only a video without any visual extras (M = 3.80). Precedents for centering can be found, e.g. in [60]. Positive values therefore indicate a positive change of attitude towards the politician from the baseline on a given dimension; negative values represent the opposite.

The main effect of the voiceovers were significant. Subjects in the condition of negative voiceover attributed less positive qualities to the politician (M = -.92, *SD* = .92) than viewers who saw material which included ambivalent voiceover (M = -.22, *SD* = 1.01), and in turn less than the subjects in the condition of positive voiceover (M = .96, *SD* = .82), *F* (2, 471) = 194.38, *p* < .001, *η*^2^ = .42.

### The impact of additional visual elements on assessments

The impact of additional visual elements (stimuli) was not consistent with the predictions, *F* (3, 471) = 15.20, *p* < .001, *η*^2^ = .05. Post-hoc tests showed that people who saw the video material with positive images found the politician positive (M = .20, *SD* = 1.12), but these ratings did not differ from the condition with negative tickers (M = .18, *SD* = 1.09). Unexpectedly, the subjects who saw positive tickers (M = -.17, *SD* = 1.18) evaluated the politician negatively, but better than in the condition of negative images (M = -.42, *SD* = 1.31); “low” (negative) and “high” (positive) results were significantly different at *p* < 0.001, but there were no differences within the low / high scores. The main effect of the additional stimuli − described more fully below − confirms H1.

We expected on the basis of H2 that images (M = .02, *SD* = 1.16) would have a stronger effect on the assessment of the politician than tickers (M = -.11, *SD* = 1.26), but differences between these combined groups checked by contrast test were not statistically significant, *t* (470.30) = 1.30, *p* = .293, therefore H2 is not supported. We assumed on the basis of H3 that negative stimuli would have a negative impact on assessment (and vice versa), but the direction of impact is ambiguous (as we noted above); although negative images are most likely to have a negative effect than any other stimuli combined together, *t* (185.09) = 3.67, *p* < .001. H3 is therefore only partially confirmed.

**Fig 1:**
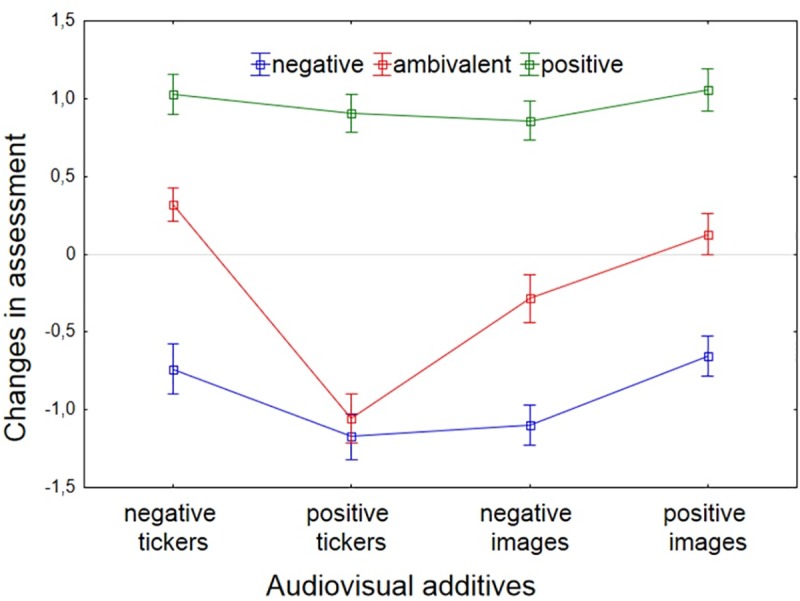
Changes in the assessment of the politician depending on additional visual elements and emotional valance of the voiceover. Whiskers represent a standard error; baseline levels are ratings centered on the average level of all attributes for all neutral conditions (see results paragraph).

The interaction between emotional valence of voiceover and visual elements (stimuli type) was statistically significant, *F* (6, 471) = 4.38, *p* < .001, *η*^2^ = .03. The direction of opinion changes for all three types of film was similar—whereas positive images and also negative tickers led to an improved assessment of the politician, positive tickers and negative images undermined this perception; the interaction was largely due to the effects of visual elements in the condition in which the subjects viewed an ambivalent voiceover. With ambivalent voiceover, the politician was evaluated as poorly with positive tickers as for the negative voiceover (see Fig 1).

### Preferred source of information and reception of politician in conditions of content inconsistency

The next analysis checked how content inconsistency between voiceover and additional visuals influenced the reception of the politician. Inconsistent content is understood as positive voiceover coverage with negative add-ons (and vice versa); affective valence of additional stimuli was expressed with visual additives to video material: positive means positive tickers or images (there were no differences between the two types of add-ons). The impact on the reception of the politician was analyzed in a 2 x 2 model.

Ambivalent voiceover conditions and a lack of additional stimuli were omitted in this analysis (ambivalence is inherently ambiguous; no add-ons sign means by assumption no inconsistencies).

No separate impact was found in stimuli sign, *F* (1, 319) = .04, *p* = .835, *η*^2^ = .00 or stimuli correspondence, *F* (1, 319) = .03, *p* = .869, *η*^2^ = .00, whereas their interaction was revealed, *F* (1, 319) = 372.90, *p* < .001, *η*^2^ = .54. When visual communication is consistent, subjects perceive the politician positively in a positive condition (M = .94, *SD* = .82) and negatively in a negative condition (M = -.91, *SD* = .90); however when add-ons were contradictory to the audio narration—subjects ignored visual addenda (tickers and images) and used base information from the voiceover—in the case of negative additions, ratings were still positive (M = .98, *SD* = .83) and vice versa (M = -.92, *SD* = .94), which means that in the situation of content inconsistency, visual additions had little impact on the assessment of the politician. Hypothesis H4 was not supported.

Given the above, it was interesting to see how subjects remembered the presented material: do they remember more information provided in the voiceover or in visual add-ons? The respondents filled in an assessment questionnaire in which they were asked to answer questions about the qualities of the politician (for example, whether he was a bad or a great student). Under the condition of contradictory information, a movie depicted the politician in a favorable light in all features, while add-ons gave negative information (or vice versa). For six consecutive questions, subjects could indicate an answer from the voiceover or the add-ons. Because answers expressing uncertainty did not provide information in the case of conflicting messages—they were omitted, but while these indicators did not show a clear degree of memorization, they did however display a preference for information sources. Preference indicators were expressed as percentages (a fraction to the number of questions). The two indicators (voiceover vs. add-ons) were treated as within factors; differences between the types of add-ons were investigated in the condition of information contradiction: a (2) x 4 mixed model was used. If a subject did not give a strictly positive or negative answer, it meant that he or she could not give a clear answer to the question; therefore, the average for the two preference indices is the percentage of confidence in the subject’s response.

A significant effect was obtained for preferences for information source, *F*(1, 158) = 271.43, *p* < .001, *η*^2^ = .59. Additional stimuli as a source of information in the condition of inconsistent information were used by subjects significantly less often (M = 8.13, *SD* = 20.03) than voiceovers (M = 63.48, *SD* = 32.94), which is contrary to the assumed hypothesis H5.

**Fig 2:**
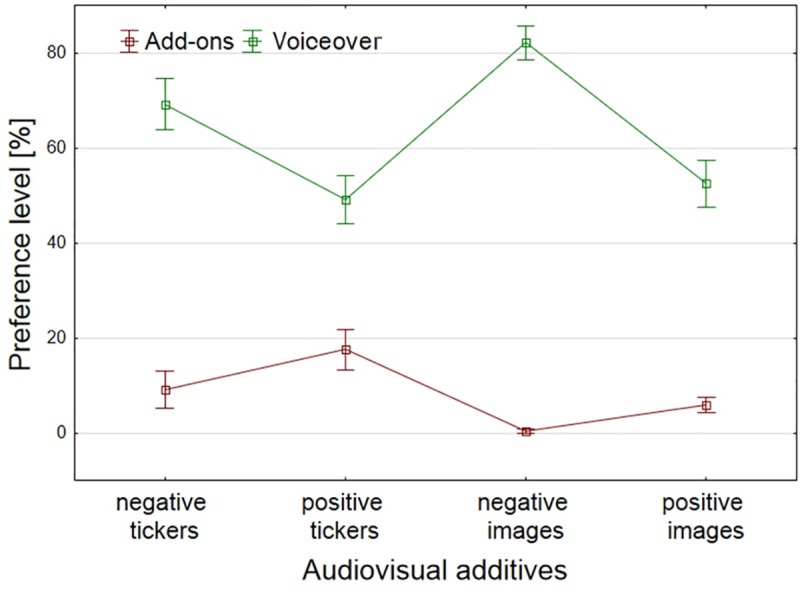
Preference of data sources in a condition of inconsistent (contradictory) information for answers to questions about the characteristics of the politician. Whiskers represent standard error.

Interaction for preferred sources and additives was also obtained, *F* (3, 158) = 10.26, *p* < .001, *η*^2^ = .07. Content preferences were quite consistent—when voiceover was negative and add-ons were positive (both tickers and images), the preference for additives increased. In the opposite situation—when a voiceover was positive and add-ons were negative, the preference for additives decreased. This may mean that subjects simply have a tendency to choose and memorize positive information. It should be noted, however, that the preferences for additive visuals were very low in each case, and that the positive results of positive tickers were indeed the highest (Fig 2).

It is worth mentioning that differences have also been observed in the type of visual add-ons, which express the degree of overall certainty of a correct answer given by the subject, *F* (3, 158) = 6.37, *p* < .001, *η*^2^ = .11. The lowest scores of certainty were obtained by subjects who were exposed to positive images (*M* = 29.17, *SD* = 14.12), while there was a slightly higher confidence level for subjects viewing positive tickers (*M* = 33.33, *SD* = 15.56) (because contradictory messages were analyzed, the content of the film itself here was negative). The highest confidence level was found for subjects in conditions of negative add-ons (tickers: *M* = 39.17, *SD* = 14.88; images: *M* = 41.27, *SD* = 10.88) (with positive voiceover) between which there were no significant differences. A contrast analysis also showed that with negative add-ons (*M* = 40.24, *SD* = 12.95), subjects were more confident with their answers than those with positive add-ons (*M* = 31.25, *SD* = 14.91, *t* (158) = -4.09, *p* < .001).

## Discussion

We hypothesized generally that additional visual elements in a video material will influence viewers’ attitudes toward a politician according to their content (H1). Specifically, we predicted that images would have a stronger effect compared to TV tickers (H2), and also that visuals with negative content would lower perceptions compared to positive content (H3). We also assumed that in the case of inconsistency of content conditions, viewers would be guided mostly by images rather than tickers and narration (voiceovers), so that the images would have the strongest impact on assessment (H4) and the viewer would choose a preferred source of information (H5).

The results confirmed only hypothesis H1. However, we have noticed a number of interesting interaction effects.

It turns out that only additional visual elements in the form of images affected the reception of the politician as we hypothesized. Tickers, by contrast, had a very weak effect. Negative images are apparently much more convincing than the text displayed on positive images and any kind of tickers. Images may be seen as an indirect form of providing data, when compared to tickers that provide direct claims. Research on indirect persuasion in advertisement has shown that this type of persuasion in the form of images is more effective than direct persuasion with the use of text [97]. It is possible that this is related to psychological reactance, which is an aversive affective state experienced whenever a person’s freedom is restricted [98]. This can occur when people see a clear message (on the ticker), which does not allow for interpretation of the described facts. Psychological reactance is one of the most prominent forms of resistance, considered as an important reason for message rejection [99]. However, images may be perceived as a subtle form of persuasion, which may overcome this reactance.

At the same time, the strongest effect was observed with negative images. This would confirm the results of previous studies showing the power of negative images, [4, 16, 100, 101]. This can also be explained by the effect of negativity, e.g. [56]. However, the tickers with negative content did not produce similar effects and even improved the reception of the politician. Perhaps negative tickers as an additional element evidently prepared by journalists were perceived as intentional attacks (see: [102]). Our material could also be seen as a political advertisement. Empirical evidence shows that negative advertising in campaigns in relation to a political opponent is not effective and people are not fond of it [103, 104]. A strong negative message about politicians can sometimes be treated as a planned strategy, part of an exaggerated negative campaign [105], which aims to undermine their image. In this situation, the source of this attack may be considered aggressive and unscrupulous [100]. As a consequence, negative political messages may also produce strong counterarguments [106–109]; information obtained from this source may be ignored by the public [110], considered unbelievable [111], and even elicit a “boomerang effect”—(change to the opposite direction from the advocated position) [112] and “backlash effect” (negative feelings towards the source of the message, without a significant impact on the attitudes towards the object of attack) [113–116]. Redlawsk [117] argues that people cannot be treated as “calculators” because their reasoning is motivated. Therefore, after being exposed to negative information about the preferred candidate, they begin to feel even more sympathetic than before receiving the data. In this way, the entire negative message becomes ineffective [113, 118, 119]. Likewise, we can explain the opposite reaction to tickers with positive content. Positive information on tickers may seem suspicious, since the overt flattering of politicians by journalists is a rather rare practice, so this may have caused the above mentioned psychological reactance. Therefore, the fact that the protagonist of the reportage was a politician could have implications for the reception of him and the entire video material.

The use of images can highlight a fragment of a particular politician’s history and can make them more cognitively available for people and more important for the recipients when making judgments [5]. In turn, the tickers may have been too unambiguous in reception, not allowing free interpretation and self-inference. Therefore, they worked oppositely on attitudes and induced the above-mentioned psychological reactance.

The above data is supplemented by the analysis made in the situation of obvious inconsistency, i.e. in conditions in which the audio narration conveyed different content than the tickers and images. It turns out that then the respondents practically ignored the additions, basing their assessments on the narrator. Rejection turned out to be particularly strong when visual additives transmitted negative content. This may also suggest a positive effect, which is particularly noticeable in the case of women [120] or among social media users [121] (and such persons undoubtedly dominated in the study sample). For example, an analysis of nearly 7,000 shared articles from the New York Times shows us that those which contained positive emotions were forwarded much more often than those containing negative emotions [121]. It turns out that also in political communication positive tweets have a greater chance of repetition than negative ones [122].

### Practical implications

The research has shown that the tickers, though they seem to be a useful way to convey shorter content, may have evoked counter-productive reactions. This, in particular, may be a warning to biased media, who may want to manipulate the viewer’s perception of disliked politicians using tickers. The results also suggest that in condition with a lack of consistency, viewers will prefer the audio channel. It seems, therefore, that they value this channel more. All this together would indicate the ineffectiveness of visual additions for shaping attitudes.

### Limitations of the study

The research seems to be the first attempt to experimentally verify the impact of tickers and images contained in a video message on the attitudes of the viewer. Applications should therefore be made with caution, especially regarding external validity. It should be noted that the tickers used in the research presented a set of assessments of a person—messages of this kind are probably not common practice in real journalistic materials.

In addition, very different ticker formats will appear in the media. For example, Brechman and colleagues [37] distinguish “crawls”, which move slowly to the left, and “flippers” which are static text lines that are updated once in a while. Information tickers can also take various graphical forms and colours. Most likely, however, it is probably very rare that the content of additional visual elements is in clear contradiction to the narration of the audio. It should also be noted that in the prepared stimulus material, the audio message was played to the respondents from the very beginning to the end of the film, and the images or tickers appeared sporadically at certain times. This may therefore constitute the reason for the audio messages being dominant.

### Future research

This study was exploratory and is in need of further replication, which could simultaneously validate the nature of the reception of tickers and images—how they are perceived, processed, and what their role in formulating evaluations are.

In subsequent studies, it would be worth asking directly whether the subjects noticed the tickers and images displayed in the video material. It is worth mentioning that people who are notified about the tickers can have a disrupted reception of messages [88]. We are also aware about the problem regarding the equivalence of the tickers and images used in our study. Photos carry much more information (emotions, associations) than tickers. This problem seems very difficult (if not impossible) to eliminate, in case of using complex pictures as stimuli. Therefore, in future studies it is worth considering using more unambiguous, simpler image stimuli or controlling the associations and the intensity of the emotional effect they are to generate.

Perhaps it would also be necessary to check the perception of additional visual elements themselves, e.g. what ticker formats and type of content should be used to evoke a particular reaction, like trust or increased interest and memorization. In future studies, it would be advisable to check whether the observed results turn out to be similar if the plot concerns a representative of a different social group as opposed to the politician (e.g. entrepreneur, student, etc.), because the label “politician” may activate a specific stereotype, especially related to the dimension of morality [96]. For the future, it may also be worth asking how the perception of physical attractiveness changes when the material contains additional affective tickers or images.

## Supporting information

S1 Data Examples of the stimulus material available here: osf.io/dzuf7. (CSV)
